# Interplay between Diffusion and Bond Cleavage Reaction for Determining Release in Polymer–Drug Conjugates

**DOI:** 10.3390/ma16134595

**Published:** 2023-06-26

**Authors:** George Kalosakas

**Affiliations:** Materials Science Department, University of Patras, GR-26504 Rio, Greece; georgek@upatras.gr

**Keywords:** controlled drug release, Monte Carlo numerical simulations, bond degradation, reaction–diffusion systems, release kinetics

## Abstract

In conjugated polymeric drug delivery systems, both the covalent bond degradation rate and the diffusion of the freely moving drug particles affect the release profile of the formulation. Using Monte Carlo simulations in spherical matrices, the release kinetics resulting from the competition between the reaction and diffusion processes is discussed. For different values of the relative bond cleavage rate, varied over four orders of magnitude, the evolution of (i) the number of bonded drug molecules, (ii) the fraction of the freely moved detached drug within the polymer matrix, and (iii) the resulting fractional release of the drug is presented. The characteristic release time scale is found to increase by several orders of magnitude as the cleavage reaction rate constant decreases. The two extreme rate-limiting cases where either the diffusion or the reaction dominates the release are clearly distinguishable. The crossover between the diffusion-controlled and reaction-controlled regimes is also examined and a simple analytical formula is presented that can describe the full dependence of the release time on the bond cleavage rate constant. This simple relation is provided simply by the sum of the characteristic time for purely diffusional release and the bond cleavage decay time, which equals the inverse of the reaction rate constant.

## 1. Introduction

Polymer matrices loaded with bioactive agents provide an efficient platform for the controlled release of their cargo, as well as suitable materials for tissue engineering applications, because they exhibit desired properties such as biocompatibility, responsiveness, versatility, etc. [1,2,3,4,5,6,7,8]. The release of drugs from these delivery systems can be determined by various mechanisms, namely drug diffusion, matrix swelling, or chemical reactions [9]. Depending on the case, a particular mechanism may be the dominant one, or different mechanisms may compete to determine the release kinetics.

A type of chemical reaction mechanism that may takes place corresponds to the cleavage of bonds that covalently link the drug molecules to the polymer chains. Such conjugated polymer–drug delivery systems have been discussed in several review articles; see, for example, [10,11,12,13,14,15]. The drug can be attached either at the end groups or at side chains of the polymeric macromolecules, with linkages of varying strength, ranging from relatively strong ester or amide bonds to more easily degradable labile linkages. These covalent bonds can be cleaved by various reactions, as for example hydrolysis, proteolysis, photolysis, oxidation or reduction, and others [11]. Therefore, the linkage degradation rate can be controlled by different factors, such as the pH of the external medium, particular enzymes located at the targeted regions of desired action, the application of irradiation, or various redox agents.

Polymer–drug conjugates are able to provide prolonged release, improved drug solubility, reduced toxicity, and other desired properties. As a result, these systems have been frequently used to load and efficiently deliver various anticancer drugs, as for example doxorubicin [16,17,18,19,20], paclitaxel [21,22,23,24], and docetaxel [25]. Further, they have been proposed as potential formulations for intravitreal injections [26]. In earlier studies, polymer conjugates containing other bioactive compounds, such as variants of vascular endothelial growth factors [27,28], model proteins like bovine serum albumin [29], or smaller molecules [30], have been demonstrated.

Theoretical models and numerical investigations can provide insights into the dependence of the release characteristics on different parameters of the system, thus facilitating the design of delivery devices that exhibit the desired release rates [9,31,32,33,34,35,36,37]. As regards conjugated polymer–drug formulations, Pitt and Schindler have discussed various properties of the release kinetics obtained through an analytical solution of the reaction–diffusion equation in a one-dimensional setting, which is appropriate for slab geometries [38]. In another work, a statistical–kinetic model describing hydrogel degradation has been extended and applied to the case of the release of proteins that are covalently attached within the matrix [39].

Monte Carlo simulations are very suitable to describe stochastic events such as those involved in chemical reactions and diffusive motions. There exist many Monte Carlo calculations applied in various drug delivery devices for different situations. However, in the majority of these cases, the release is determined mainly by diffusion and/or matrix bulk degradation or erosion [40,41,42,43,44,45,46,47,48,49,50,51,52,53,54,55,56,57,58,59]. Corresponding applications in conjugated polymer–drug systems containing labile bonds and the subsequent diffusion of the detached drug particles are rare [60].

Here, Monte Carlo numerical simulations are carried out in polymer matrices with a spherical geometry in order to address the competition between bond cleavage rates and diffusion. The chemical reaction rate constant is varied over a few orders of magnitude and the resulting effects in the evolution of the attached (bonded) versus detached drug molecules within the matrix, as well as the corresponding release profiles, are calculated. Both reaction-controlled and diffusion-controlled regimes, as well as their crossover region, are examined. The characteristic release time scales are quantified and it is found that they can be substantially prolonged by several orders of magnitude as the bond degradation rate constant decreases.

## 2. Numerical Methods

The numerical calculations are performed on a cubic lattice of size equal to the diameter of the simulated spherical matrix. Within this lattice, a polymer spherical region of radius R is defined. This radius is given in units of the lattice constant $l_{u}$, which constitutes the unit of length in our system. Inside the sphere, a number of drug particles are randomly placed, according to the desired concentration $C_{0}$ ($0<C_{0}\leq 1$); if $M_{S}$ is the total number of lattice sites inside the sphere, then the $C_{0}\cdot M_{S}$ of them are randomly occupied initially by drug particles. Double occupancy is forbidden due to excluded volume interactions.

At the beginning of each simulation, all drug molecules are covalently linked to the polymer matrix. The bond cleavage reaction is described through the corresponding rate constant parameter $k_{b}$ ($0<k_{b}\leq 1$). During the simulation, at each Monte Carlo step, a drug particle is chosen at random. If the particle is bonded to the matrix, then a random number is drawn from a homogeneous distribution in the interval 0 to 1. When this random number is smaller than the particular value of the parameter $k_{b}$, then the corresponding bond breaks and the chosen particle is no longer attached to the matrix; therefore, this particle can subsequently freely diffuse. Otherwise, the chosen drug molecule remains bound in its initial position.

The diffusion of the detached, freely moving drug particles is simulated using the Monte Carlo procedure that is described in Section 2.1 of Ref. [49]. In brief, if during the simulation the randomly chosen drug particle is detached from the polymer matrix and it is free to diffuse, then one of its six neighboring lattice sites is randomly selected (assigning equal probabilities for all six possible directions of movement). If the selected neighboring site is empty, then the particle moves to this new site. Instead, if the neighboring site is already occupied, then the particle stays in its initial position. When a freely moving detached molecule during its random diffusion exits the boundaries of the spherical region of radius R, then it is released to the external environment and removed from the simulation. In this case, the number of drug particles within the polymer is decreased accordingly.

During the simulation, at each Monte Carlo step, when a drug particle is randomly picked up, either bound or detached, the Monte Carlo time is increased by the quantity $1/N\left(t\right)$, where $N\left(t\right)$ is the total number of drug molecules still remaining inside the polymeric matrix at time t (regardless of whether they are conjugated or not). Therefore, the unit of time $t_{u}$ in our simulations is given by the mean time needed by a drug molecule to move by a distance $l_{u}$ (see also the relevant discussion in Ref. [49]). As a result, the considered values of the relative cleavage rate constant $k_{b}$ mentioned above are given in units of $t_{u}^{-1}$.

At the beginning of each simulation there exist $N\left(t=0\right)\equiv N_{0}=C_{0}\cdot M_{S}$ drug molecules within the polymer matrix. $M_{S}$ is uniquely determined by the radius R of the sphere. The number of drug particles inside the sphere, $N\left(t\right)$, is recorded as a function of time, along with the number of covalently bonded molecules $N_{b}\left(t\right)$, until the end of the simulation, when all drug molecules are released from the formulation and $N\left(t\right)$ vanishes. The number of released drug molecules at time t equals $N_{0}-N\left(t\right)$, while the amount of fractional release, which provides the experimentally relevant release profile, is $1-N\left(t\right)/N_{0}$. Every Monte Carlo simulation is repeated 100–300 times using the same parameter values but different random number sequences (resulting, for example, in different initial distributions of the drug particles inside the matrix and different sequences of the subsequent events), in order to obtain statistical averages over the different realizations.

This work focuses on the effects of the cleavage reaction rate $k_{b}$ on the release characteristics. Therefore, the other system parameters are kept fixed here: the radius of the polymer matrix has a value R=20 and the initial drug load is $C_{0}=0.5$ (i.e., the $M_{S}$ lattice sites within the considered spherical region are initially half-filled). The variation of the bond cleavage rate $k_{b}$ spans four orders of magnitude, ranging from 0.0001 up to its maximum value of 1.

## 3. Results and Discussion

First, results are presented for the evolution of the bonded drug particles in Section 3.1 and the fraction of the detached drug molecules over the total number of molecules within the polymer sphere in Section 3.2, as obtained by the Monte Carlo calculations. Then, the influence of the reaction constant parameter $k_{b}$ on the resulted drug release profiles is discussed in Section 3.3. Finally, the characteristic time scale of release is quantified in Section 3.4 and its dependence on the cleavage rate constant is analyzed.

### 3.1. Cleavage Reaction: First-Order Kinetics

As already mentioned, the time dependence of the number of conjugated drug particles $N_{b}\left(t\right)$ is monitored during the numerical simulations. This number starts from the initial value $N_{b}\left(t=0\right)=N_{0}$, since all drug molecules are bonded in the polymer chains at the beginning of each simulation, and it gradually decreases as more and more particles are detached. At some point, $N_{b}$ vanishes during the simulation; this condition is necessary before the release of all drug molecules from the matrix, signifying the end of the simulation.

**Figure 1:**
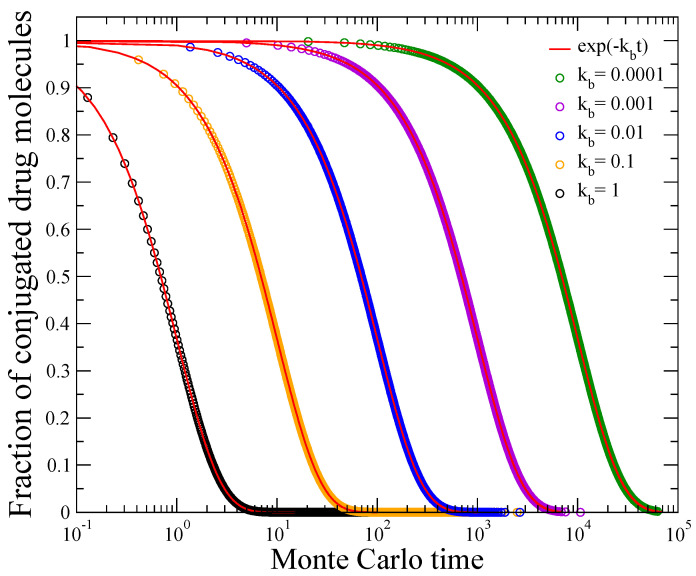
Evolution of the fraction of covalently bonded molecules $N_{b}\left(t\right)/N_{0}$ obtained by the Monte Carlo simulations (circles), for different values of $k_{b}$ as indicated in the plot. Solid red lines show the corresponding first-order kinetics formula $\exp\left(-k_{b}t\right)$, Equation (1).

Figure 1 shows results for the fraction of covalently bound molecules over their initial value, i.e., for the quantity $N_{b}\left(t\right)/N_{0}$. Circles of different colors correspond to the Monte Carlo data for different values of the cleavage rate constant: $k_{b}=1$ (black), $k_{b}=0.1$ (orange), $k_{b}=0.01$ (blue), $k_{b}=0.001$ (violet), and $k_{b}=0.0001$ (green). Obviously, the smaller the $k_{b}$, the more delayed the drug detachment. Along with the numerical data for different values of $k_{b}$ (circles), the continuous red lines depict the corresponding result obtained by assuming a first-order cleavage reaction:

(1) $$N_{b}\left(t\right)/N_{0}=e^{-k_{b}t}$$

The perfect agreement of Equation (1) with the Monte Carlo results, for the whole evolution up to the detachment of all drug particles within the formulation, reveals that the bond cleavage chemical reaction, simulated as discussed above, exhibits first-order kinetics. Such first-order kinetics of $N_{b}/N_{0}$, described by Equation (1), hold also for all other examined values of the relative rate constant parameter $k_{b}$, between those presented in Figure 1.

### 3.2. Fraction of the Freely Diffusing Molecules within the Polymer Matrix

The number of the detached, free moving drug molecules within the formulation, $N_{f}$, is given at any time from the difference $N_{f}\left(t\right)=N\left(t\right)-N_{b}\left(t\right)$. This number starts from zero, $N_{f}\left(t=0\right)=0$, because all molecules are bonded initially, and it then increases as the linkages are sequentially cleaved, and finally drops back to zero as the diffusing drug particles are released.

**Figure 2:**
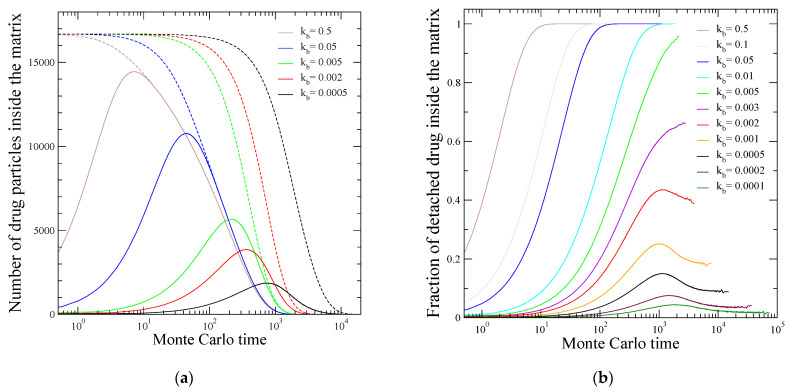
Evolution of the population of detached drug particles, which are free to diffuse within the polymer matrix, for various values of the bond cleavage rate $k_{b}$. (**a**) Continuous lines show the number of free drug particles $N_{f}\left(t\right)$ and dashed lines show the total number $N\left(t\right)$ of drug particles (bonded and free) within the formulation. Lines of the same color correspond to a particular value of $k_{b}$ as indicated in the figure. (**b**) Fraction of the detached drug molecules over the total number of both bonded and detached drug particles, $N_{f}\left(t\right)/N\left(t\right)$, inside the polymer matrix.

Figure 2a presents the time dependence of the freely moving particles $N_{f}\left(t\right)$ (solid lines), along with the total number of drug molecules—both bonded and freely moving—$N\left(t\right)$ (dashed lines) within the formulation, for different values of $k_{b}$. We can see the initial increase and the subsequent drop in $N_{f}\left(t\right)$, as well as the continuous decline in $N\left(t\right)$. For relatively fast reaction rates (larger values of $k_{b}$ in Figure 2a), the $N_{f}\left(t\right)$ and $N\left(t\right)$ curves diminish together, since, after some point, the whole drug population within the polymer consists of freely moving molecules due to the rapid degradation of the linkages. The larger the $k_{b}$, the earlier the increase in $N_{f}\left(t\right)$ and the higher its maximum value, and the wider the region of the identical drop in the $N_{f}\left(t\right)$ and $N\left(t\right)$ lines. In this case, diffusion is the rate-limiting mechanism for the drug delivery system. However, as the cleavage rate becomes smaller and smaller, this situation gradually changes. For the smaller value of $k_{b}$ depicted in Figure 2a, one sees that the free drug population drops to vanishing values, while there are still many drug particles present in the matrix. The latter remain conjugated to the polymer chains. In this case, bond cleavage is so slow that the chemical reaction constitutes the rate-limiting step determining the characteristic time scale of drug release from the formulation.

Figure 2b demonstrates the crossover between the two limiting cases of diffusion-controlled and reaction-controlled regimes, showing the evolution of the ratio of free drug particles over the total number of drug particles within the polymer, $N_{f}\left(t\right)/N\left(t\right)$. For fast cleavage rates ($k_{b}>0.01$), the fraction of detached drug reaches unity and, of course, it remains there until the release is completed. The higher the reaction rate, the sooner the $N_{f}\left(t\right)/N\left(t\right)$ becomes equal to 1. This constitutes a diffusion-controlled release. In the opposite limit of reaction-controlled delivery ($k_{b}\sim 10^{-4}$), the percentage of free moving drug within the matrix remains at small levels. It exhibits a more or less evident, transient peak before reaching an almost steady state at relatively low values. The crossover regime between these two limiting cases is revealed by the behavior of the fraction of freely diffusing drug shown at cleavage rates $k_{b}\sim 10^{-3}$. This intermediate regime seems to be characterized by the absence of a steady state behavior, after some point, of the ratio $N_{f}\left(t\right)/N\left(t\right)$. This is in contrast to the obtained steady state at unity, or at low values, when diffusion or reaction, respectively, is the dominant release mechanism.

### 3.3. Drug Release Profiles

The fractional release of a drug from a delivery device is a quantity that is usually observed experimentally, where the amount of released drug at time t divided by the total amount of drug contained initially in the formulation is plotted as a function of time. In the used Monte Carlo calculations, drug release profiles are provided by the evolution of the variable $1-N\left(t\right)/N_{0}$ (see Section 2). Here, it is shown how the drug release profiles are affected by the variation of the chemical reaction rate constant $k_{b}$, considering the transition from the diffusion- to the reaction-controlled regime.

**Figure 3:**
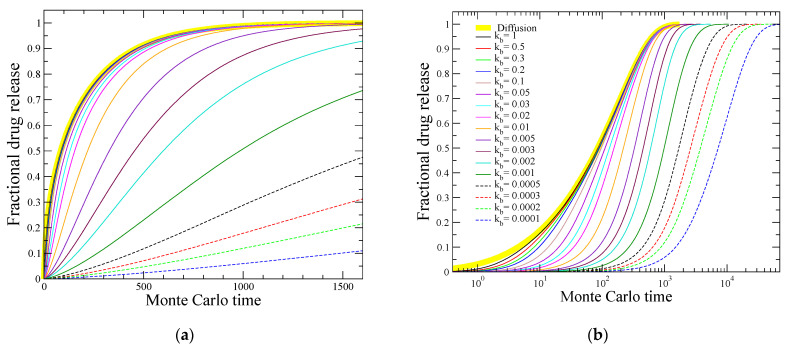
Drug release profiles shown in (**a**) linear–linear and (**b**) linear–log plot, for various values of the relative cleavage rate parameter $k_{b}$ as indicated in the figure. The thick yellow line at the left of each plot presents the fractional drug release resulting from an identical spherical matrix where there exists only diffusion, i.e., there are no bonded drug particles and all molecules are free to diffuse from the beginning of the simulation.

Figure 3 depicts the obtained fractional drug release for all the different values of $k_{b}$ considered in this work. In order to compare with the case where there is not any chemical reaction but simply diffusion, it is also plotted the release obtained by a spherical matrix of the same size and the same initial drug load, where there is no bonded drug initially and all particles are freely moving from the beginning of the simulation (yellow thick lines in Figure 3).

It can be seen from Figure 3 that for the larger values of $k_{b}$, the release profiles are almost identical to the case where there is merely diffusion; the corresponding curves are not distinguishable in the linear–linear plot (Figure 3a), while only small differences at the beginning of the release are observed in the linear–log plot (Figure 3b), as expected due to the short time needed for the detachment of the drug molecules. This is consistent with the previous discussion regarding the diffusion-controlled release.

The situation changes when decreasing the parameter $k_{b}$, as indicated by a gradual delay in the release. Figure 3b shows that for $k_{b}\leq 0.01$, the release profiles are distinguishable among one another until the end of the release process, and they are shifted to longer times as the cleavage rate becomes slower.

It seems that just the inspection of the release profiles of Figure 3 cannot provide a clear distinction between the reaction-controlled regime and the intermediate crossover regime. Instead, the case where diffusion is dominant can be easily recognized because the release curves coincide to a large extent in this regime. In contrast, as one proceeds from the intermediate to the reaction-controlled regime, gradual changes seem to occur in the drug release profiles. However, quantitative aspects of the fractional release kinetics are able to distinguish these regimes, as shown in the next subsection.

### 3.4. Characteristic Release Time Scales

In order to quantify the characteristic time scale of a release profile and then explore its variation with $k_{b}$, two methods are employed here to calculate the average times, which provide similar results.

In the first case, an average release time is obtained through the numerical integration of the fraction $N\left(t\right)/N_{0}$ of drug particles remaining inside the polymer matrix. This quantity changes from 1 to 0 during the release and it is complementary to the fractional release, as their sum is always equal to 1. Its integration up to the end of the release provides a direct estimate of the average time of the process.

In the second case, the characteristic time scales are derived through fittings of the release profiles with stretched exponential functions. A stretched exponential (or Weibull) function represents a relatively simple formula that is frequently used to describe release profiles [41,49,53]. Then, the average release time is obtained by the values of the fitting parameters, as discussed in Appendix A (see Equation (A2) below).

**Figure 4:**
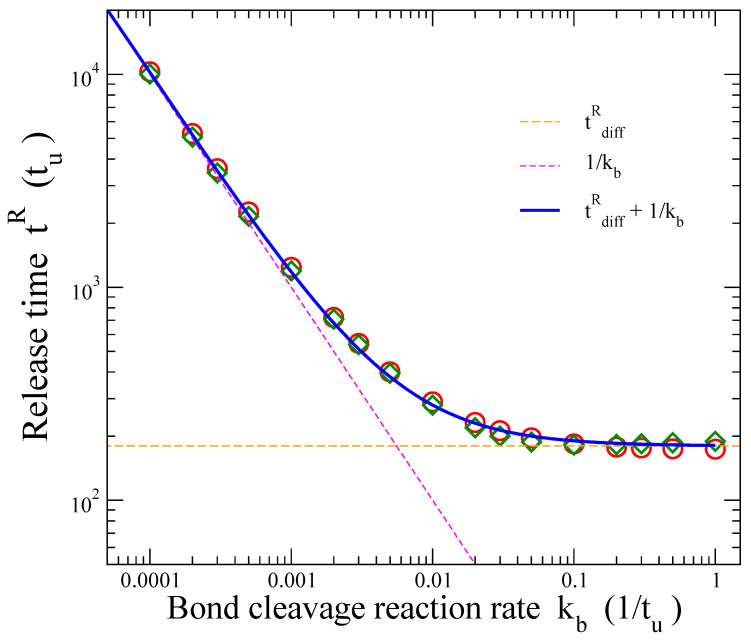
Dependence of the characteristic release time $t^{R}$ on the bond cleavage rate constant $k_{b}$. Green diamonds represent the average release times obtained through fittings of the release profiles with stretched exponentials (see Appendix A). Red circles show the release times derived through direct integration of the fraction of drug particles inside the matrix. The horizontal dashed line corresponds to the value $t_{diff}^{R}$ of the average release time for a purely diffusional process, without bonded drug particles initially. The inclined dashed line is the graph of the bond cleavage decay time $t_{reac}=1/k_{b}$. Solid blue line represents their sum, Equation (2).

Figure 4 presents the variation of the characteristic release time $t^{R}$ with $k_{b}$. Diamonds and circles depict the numerical results obtained using the two methods mentioned above, leading to similar outcomes. The two dashed lines shown in the plot represent the value of the diffusional release time $t_{diff}^{R}$ (horizontal line) and the characteristic decay time of the cleavage reaction given by $t_{reac}=1/k_{b}$ (inclined straight line). The former is the average time of the fractional release when there is solely diffusion, corresponding to the release profile shown by the thick yellow line in Figure 3. The latter formula provides the average time of the first-order kinetics of the chemical reaction.

It can be seen from Figure 4 that for the larger values of the cleavage rate constant $k_{b}$, the release times coincide with the characteristic time $t_{diff}^{R}$ of purely diffusional release, while, for the smaller values of $k_{b}$ considered here, the release times converge to the average time of the chemical reaction $t_{reac}=1/k_{b}$. The former case corresponds to diffusion-controlled release and the latter to reaction-controlled release. These two limiting regimes, as well as the smooth crossover from one to the other, are clearly observed in Figure 4.

It is tempting to test whether by merely considering the algebraic sum $t_{diff}^{R}+t_{reac}$ of these two limiting behaviors, one would be able to describe the numerically obtained results for the release time of the polymer–drug conjugated system for all different values of $k_{b}$. Indeed, the blue curve in Figure 4 represents the sum of the two limiting behaviors:

(2) $$t^{R}=t_{diff}^{R}+\frac{1}{k_{b}}$$

This simple analytical relation seems to describe well the full dependence on $k_{b}$ of the characteristic release time obtained by the Monte Carlo simulations in this work, including the intermediate crossover regime. When diffusion (or reaction) is the rate-limiting mechanism, then the first (or the second, respectively) term of the sum dominates.

A final remark regarding Figure 4 is that while the value $k_{b}=1$ constitutes the upper limit for the bond cleavage rate constant in the implemented Monte Carlo scheme, there is not any finite lower limit: $k_{b}$ can take any positive value (≤1), however small it is, depending on the strength of the covalent linkage attaching the drug particles to the polymer matrix. The stronger is the bond, the lower is the rate $k_{b}$. Figure 4 demonstrates that for the smaller values of $k_{b}$, when the reaction becomes the dominant release mechanism, the characteristic release times coincide with the decay times of the first-order cleavage kinetics. Thus, the release time from a polymer–drug conjugate delivery system can be increased unbounded by orders of magnitude as the covalent linkage becomes stronger. On the contrary, there exists a lower limit for the release time, set by diffusion; regardless of how weak is the linkage of the drug to the polymer, the characteristic release time of the system cannot be smaller than the diffusion-limited value $t_{diff}^{R}$.

This study has focused on the effects of the cleavage reaction parameter $k_{b}$ on the release from polymer–drug conjugates. Thus, the other parameters of the formulation have remained fixed. Based on the results obtained here, it is expected that the dependence of the characteristic release time on the other system parameters (such as the radius of the spherical matrix, the initial drug load, and the drug diffusion coefficient) is mediated by their effects on the time $t_{diff}^{R}$ of the corresponding purely diffusional release. The latter effects have been fully quantified in Ref. [49]. In other words, Equation (2) is anticipated to also hold when the previously mentioned parameters are varied, but the quantity $t_{diff}^{R}$ is changed accordingly [49]. This will be verified in a future work.

## 4. Conclusions

Drug release from conjugated polymer–drug matrices is investigated here, using Monte Carlo numerical simulations. The influence of the cleavage reaction rate constant on the behavior of the system is addressed. Apart from typical drug release profiles, the evolution of the fraction of the bonded drug molecules, as well as the number and the percentage of the freely diffusing particles within the formulation, are also presented. The cleavage reaction exhibits first-order kinetics.

Signatures of the two extreme rate-limiting regimes, i.e., the diffusion-controlled and the reaction-controlled release, respectively, as well as of the intermediate crossover regime between one and the other, are clearly manifested in two quantities. These are (i) the dynamics of the percentage of the detached, freely moving drug within the polymer formulation and (ii) the dependence of the characteristic release time on the cleavage rate constant.

In the former case, it has been found that the fraction of the detached drug particles over the total number of drug particles (bonded and detached) within the matrix exhibits, after some time, a steady state behavior at 1, or at a very low value, when the diffusion or the reaction, respectively, is the rate-limiting mechanism. In the crossover regime, such a steady state seems to be absent.

In the latter case, the characteristic time scale of the release in the diffusion-controlled regime tends toward the value characterizing a purely diffusional release process, which sets a lower limit in the release time of the conjugated system. In the opposite limit of the reaction-controlled regime, the characteristic release time scale follows the unbounded $1/k_{b}$ relation, set by the first-order bond cleavage decay. It has been found that a simple interpolation sum of these two limiting behaviors provides an accurate description of the characteristic time scale of the release for the whole range of the reaction rate constant parameter $k_{b}$.

Inspecting the fractional release dynamics for various values of $k_{b}$ unveils only the regime dominated by diffusion, where the release profiles tend to coincide and are indistinguishable between each other. However, one is not able to discriminate the reaction-controlled regime from the intermediate crossover region by just looking at the release profiles, because there are gradual changes in the release curves as $k_{b}$ is varied in this region.

The presented model is applicable to any type of polymer–drug conjugated system as long as the following two conditions are met: (i) polymer swelling and erosion or bulk degradation of the polymeric network are not significant, but only the covalent bonds linking the drug molecules are degradable, and (ii) the cleavage reaction of these labile bonds follows first-order kinetics. The specific physicochemical properties of the drug molecule and the polymer matrix will be represented in the value of the corresponding drug diffusion coefficient. Temperature, pH, or other external factors are introduced through their effects on the cleavage rate constant and the drug diffusion coefficient.

When surface erosion or bulk degradation of the polymeric network also takes place, then the used Monte Carlo scheme can be properly adapted to describe these situations. For example, in the former case, randomly chosen lattice points at the external boundary of the formulation could be gradually removed, while, in the latter case, the drug diffusion coefficient could be increased over time in response to the degradation of the macromolecular network. These investigations are left for future studies. Further, in case that the reaction kinetics are second-order [61], or of any other type, the bond cleavage description in the used Monte Carlo scheme should be modified accordingly.

## Figures and Tables

**Figure A1:**
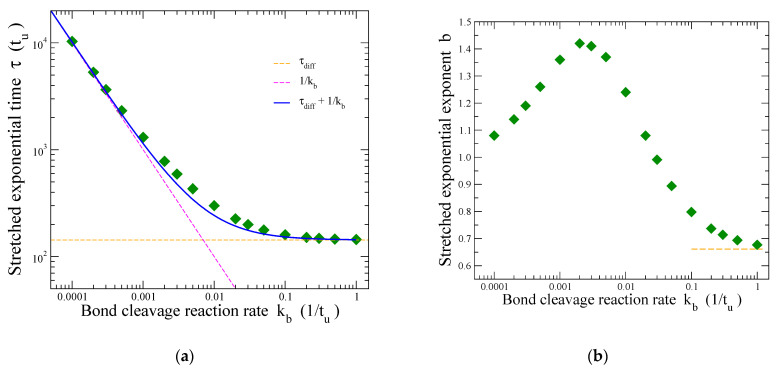
Stretched exponential parameters obtained through fittings of the release profiles shown in Figure 3 with the function of Equation (A1). Diamonds plot the dependence of (**a**) the time parameter τ and (**b**) the exponent b, on the cleavage rate constant $k_{b}$. In (**a**), the horizontal dashed line corresponds to the value $\tau_{diff}$ of the same stretched exponential fitting parameter obtained in the case of a purely diffusional release (containing no bonded drug particles initially), the inclined dashed line represents the relation $1/k_{b}$, and the continuous blue curve represents their sum $\tau_{diff}+1/k_{b}$. The horizontal dashed line in (**b**) shows the corresponding fitting value of the stretched exponential exponent when only freely diffusing particles are initially present in the matrix.

## Data Availability

The data presented in this study are available on request from the author.

## Appendix A

This appendix details how the average release times are calculated through fittings of the fractional release profiles with the stretched exponential function of the form

(A1) $$\mathrm{Fractional}\;\mathrm{release}\;\equiv \;1-\frac{N\left(t\right)}{N_{0}}=1-\exp\left[-{\left(t/\tau \right)}^{b}\right]$$

A stretched exponential function contains only two parameters: the time parameter τ and the dimensionless exponent b. Through fittings of the release profiles with Equation (A1), the values of the fitting parameters τ and b are obtained (see Figure A1 below). Then, the average time of the stretched exponential time dependence is obtained by the parameters τ and b using the formula

(A2) $$\tau_{av}=\frac{\Gamma \left(1/b\right)}{b}\cdot \tau$$

where Γ is the gamma function [49].

Diamonds in Figure A1 demonstrate the dependence of the fitting parameters τ and b on the reaction rate constant $k_{b}$. These data have been obtained by fitting the release profiles presented in Figure 3 with the stretched exponential function. Then, these values have been inserted into Equation (A2) in order to evaluate the average release times $t^{R}$ shown by the open green diamonds in Figure 4 of the main text.

From Figure A1a, it can be seen that the stretched exponential time parameter τ exhibits a similar dependence on $k_{b}$ as that shown in Figure 4 for $t^{R}$. For the larger values of $k_{b}$, the data converge to the corresponding parameter obtained in the case of sole diffusion (represented by the horizontal dashed line in Figure A1a). On the opposite limit, the data converge to the first-order reaction kinetics time scale $1/k_{b}$ (plotted by the inclined straight dashed line in Figure A1a). The blue solid line represents the sum of these two limiting behaviors, as in Figure 4.

Figure A1b presents the dependence of the exponent fitting parameter b. For $k_{b}=1$, it approaches the corresponding value obtained from a purely diffusional release (horizontal dashed line in Figure A1b). Then, it exhibits a non-monotonic behavior as $k_{b}$ decreases: the exponent b initially rises, crossing the unity in the intermediate crossover regime, and then it starts to decline when the system enters the reaction-controlled regime. For the smaller rate constants $k_{b}$ considered here, it seems that b tends to values around unity. Noticing that when b=1, the stretched exponential of Equation (A1) reduces to a simple exponential function, this behavior seems to be in accordance with the fact that the release profile in the reaction-controlled regime coincides with the first-order kinetics of the chemical reaction.
